# Optimization of PET protocol and interrater reliability of ^18^F-PSMA-11 imaging of prostate cancer

**DOI:** 10.1186/s13550-020-0593-7

**Published:** 2020-02-24

**Authors:** Sarah Piron, Kathia De Man, Vanessa Schelfhout, Nick Van Laeken, Ken Kersemans, Eric Achten, Filip De Vos, Piet Ost

**Affiliations:** 1 Laboratory of Radiopharmacy, Ghent University, Ottergemsesteenweg 460, 9000 Ghent, Belgium; 2 Department Nuclear Medicine, Ghent University Hospital, Ghent, Belgium; 3 Department Radiation Oncology, Ghent University Hospital, Ghent, Belgium

**Keywords:** PSMA, ^18^F-PSMA-11, PET/CT, Prostate cancer, Scan protocol, Interrater reliability, Acquisition time, Randomized clinical trial

## Abstract

**Background:**

Several scan parameters for PET imaging with ^18^F-PSMA-11 such as dosage, acquisition time and scan duration were evaluated to determine the most appropriate scan protocol, as well as the effect of furosemide administration on lesion visualization. Forty-four patients were randomly assigned to a dosage group (2.0 ± 0.2 or 4.0 ± 0.4 MBq/kg ^18^F-PSMA-11). All patients received a full-body PET/CT 1 h and 3 h after radiotracer injection with a scan duration of 3 min/bed position. For comparison of the scan duration, images were reconstructed for 1.5 and 3 min/bed position. Patients were intravenously administered 0.5 mg/kg furosemide with a maximum dose of 40 mg. To evaluate the furosemide effect, 22 additional patients were recruited and received one full-body PET/CT 1 h after administration of 2.0 ± 0.2 MBq/kg ^18^F-PSMA-11 with a scan duration of 3 min/bed position. To this group, no furosemide was administered. Images were scored on image quality using a 7-point scale and each suspicious lesion was described. To assess interrater reliability, two nuclear physicians scored all scans independently and described all observed suspicious lesions.

**Results:**

The 4 MBq/kg group received for all reconstructed images (60 min p.i., 1.5 and 3 min/bed position and 180 min p.i., 1.5 and 3 min/bed position) the highest median image quality score compared to the 2 MBq/kg group (*p* values < 0.01). When comparing all reconstructed images, the highest image quality score was given to images at 60 min p.i., 3 min/bed position for both dosage groups (score 5 and 6 for 2 and 4 MBq/kg, respectively). The addition of furosemide administration decreased the interference score with one point (*p* = 0.01106) and facilitated the evaluation of lesions in proximity to the ureters. The interrater reliability for the comparison of each lesion separately after more than 40 ^18^F-PSMA-11 scan readings showed an increasing *κ* value from 0.78 (95% CI, 0.65–0.92) to 0.94 (95% CI, 0.87–1).

**Conclusion:**

Although the results indicate an administered activity of 4.0 ± 0.4 MBq/kg, preference will be given to 2.0 ± 0.2 MBq/kg due to the small difference in absolute score (max 1 point) and the ALARA principle. For evaluation of lesions in proximity to the ureters, the co-administration of a diuretic can be useful. The increase of the *κ* value from 0.78 to 0.94 suggests a learning curve in the interpretation of ^18^F-PSMA-11 images.

**Trial registration:**

Clinicaltrials.gov, NCT03573011. Retrospectively registered 28 June 2018

## Introduction

In recent years, prostate-specific membrane antigen (PSMA) has been the most widely studied target for imaging of recurrent and metastatic prostate cancer. ^18^F-PSMA-11, a fluorine-18 derivative of the frequently used ^68^Ga-PSMA-11 PET radiotracer, was developed [1, 2], automatized [3] and evaluated for safety, biodistribution and dosimetry in a previously published study [4]. Before comparing the clinical efficacy of ^18^F-PSMA-11 to other PSMA tracers, a Phase 2 trial should be conducted to determine the scan protocol which will be applied in following studies and clinical practice. Phase 2 trial designs are usually exploratory where multiple scan parameters are tested to determine the optimal scan protocol. These parameters include radiotracer dosage, start of PET acquisition post injection, scan duration, image reconstruction parameters and updating the safety database. This requires the selection of the appropriate patient population. Finally, the variability between observers should be evaluated as the correct interpretation of PET images is a crucial step in the validation of the clinical efficacy of ^18^F-PSMA-11 and should lead to the development of criteria for image evaluation. The optimized scan protocol can then be applied in Phase 3 clinical trials to obtain data necessary for approval for application of the radiotracer in clinical use. In these larger studies, it is possible to evaluate the effect of the specific activity and the radionuclide on image quality, as well as the amount of nonradioactive ligand and carrier [5, 6]. The aim of this study was to determine the optimal scan protocol with regard to radiotracer dosage, acquisition time and duration and co-administration of furosemide. As a secondary objective, the interrater reliability was assessed.

## Materials and methods

All procedures performed in this study involving human participants were in accordance with the ethical standards of the Ethics Committee of the Ghent University Hospital (2017/1294) and with the 1964 Helsinki Declaration and its later amendments or comparable ethical standards (EudraCT nr, 2017-003461-96). The study was supported by the Flemish Foundation FWO TBM (T001517).

### Patients

In total, 66 patients (age 46–84 years, median 70.5) with primary staging or biochemical recurrence after curative treatment (prostatectomy with or without lymphadenectomy or radiotherapy) were prospectively included during a consultation with their treating physician. Patients who were under the age of 18 years old, who refused to be informed about accidental findings on scans and who were physically or mentally unfit to perform the sequential procedures were excluded from the trial, as well as patients suffering from heart failure with an ejection fraction < 45% and patients with a known history of anaphylactic shock after administration of CT contrast. Written, dated and signed informed consent was obtained from all patients before any trial-related procedures were conducted. In total, four patients dropped out of the study.

### Safety monitoring

All adverse events were actively monitored from time of radiotracer injection until completion of the second PET/CT scan and reported according to the CTCAE 4.0 scoring system. Although there was only one patient with an increased creatinine level in the previously conducted Phase 1 study, we deemed it useful to further evaluate the creatinine level changes before and after radiotracer injection in Phase 2. Therefore, a 3-mL blood sample was taken and compared to the latest available creatinine lab value before inclusion in the study.

### Study protocol

**Fig. 1:**
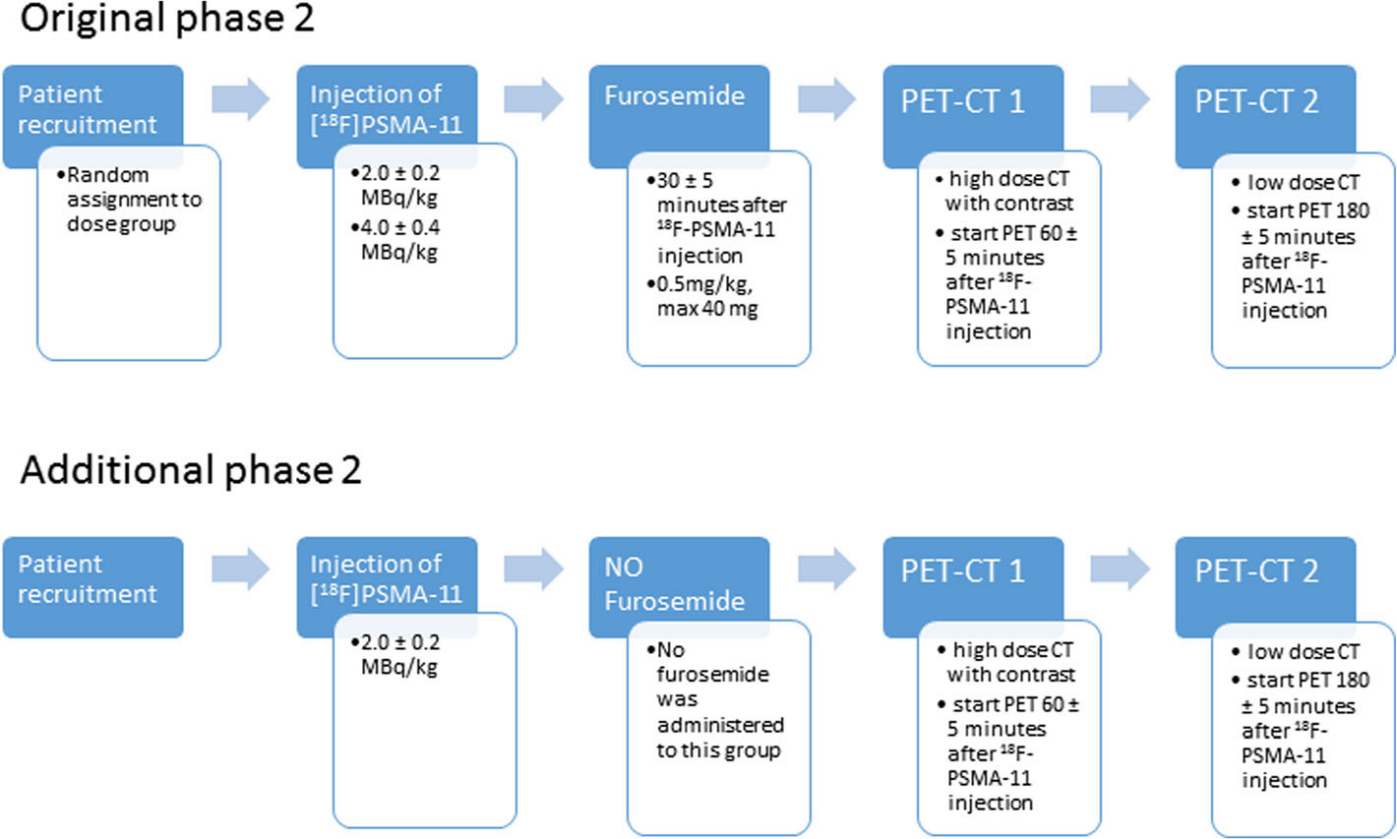
Flowchart of the study procedures

Randomization was performed using a block design with variable block sizes of two, four, and six. The first 44 patients were randomly assigned to either of the two dosage groups, 2.0 ± 0.2 MBq or 4.0 ± 0.4 MBq ^18^F-PSMA-11 per kilogram body weight. The overview of all performed study procedures is summarized in Fig. 1. Thirty minutes after receiving the appropriate dosage, each patient was given an intravenous bolus injection of 0.5 mg/kg body weight furosemide with a maximum dose of 40 mg to improve diuresis. Two whole-body PET scans were acquired at 60 ± 5 min (T60) and 180 ± 5 min (T180) post injection (p.i.) and 3 min per bed position (bp). The first PET scan was preceded by a diagnostic CT scan with administration of Visipaque® CT contrast, the second PET scan was preceded by a low-dose CT scan for attenuation correction. To evaluate the effect of furosemide on the image quality, 22 additional patients were recruited who received only one PET scan 60 ± 5 min after administration of 2.0 ± 0.2 MBq/kg ^18^F-PSMA-11 accompanied by a diagnostic CT scan. This group received no furosemide. PET/CT imaging was performed using a GE Discovery MI 3-ring system, a digital PET/CT scanner with SiPM-based PET detectors coupled to Luthetium-based scintillators, a measured resolution of around 4.5 mm and an axial field of view of 15 cm. Reconstruction of the PET scans was performed using the QClear algorithm (GE Healthcare), a block sequential regularized expectation-maximization algorithm. The reconstruction includes time-of-flight information (resolution of 290 ps FWHM), point spread function compensation, CT-based attenuation and scatter correction and a beta-parameter of 600. Each PET image was reconstructed for emission times of 1.5 min/bp and 3 min/bp to evaluate the effect of the scan duration. The individual characteristics of patients who completed all described procedures are presented in Table 1. Chi-square and one-way ANOVA was performed for categorical and continuous variables, respectively. Patient characteristics such as weight and previous undergone procedures and therapies were not accounted for in the comparison as the aim of this study was to provide a general recommendation for ^18^F-PSMA-11 imaging.

**Table 1:** Patient characteristics of patients who completed all planned procedures. Chi-square and one-way ANOVA was performed for categorical and continuous variables, respectively

	2.0 ± 0.2 MBq/kg with furosemide	2.0 ± 0.2 MBq/kg without furosemide	4.0 ± 0.4 MBq/kg with furosemide	
Number of patients	21	21	20	
Age (median, Q1–Q3)	71 (65–74)	70.5 (63.5–77.25)	69 (63–74)	*F*(2,59) = 0.149, *p* = 0.862
Weight (mean ± SD)	85.1 ± 14.4	80.9 ± 10.9	81.5 ± 11.9	*F*(2,59) = 0.691, *p* = 0.505
Staging				*Χ*^2^(2, *N* = 62) = 6.6633, *p* = 0.03573
Initial staging	0/21	6/21	4/20	
Restaging	21/21	15/21	16/20	
PSA (ng/mL) (mean ± SD)	6.90 ± 9.89	9.22 ± 18.38	7.35 ± 10.96	*F*(2,59) = 0.161, *p* = 0.851
Gleason score				*Χ*^2^(4, *N* = 62) = 2.8402, *p* = 0.5849
≤ 7	8/21	8/21	9/20	
8	4/21	8/21	5/20	
≥ 9	9/21	5/21	6/20	
Previous treatment				*Χ*^2^(8, *N* = 62) = 6.7322, *p* = 0.5658
Prostatectomy				
Salvage therapy	13/21	12/21	10/20	
Definitive	13/21	9/21	5/20	
Radiotherapy	6/21	3/21	7/20	
Current ADT	8/21	3/21	2/20	
Chemotherapy	1/21	1/21	0/20	

### (Semi-)quantitative analysis of dosage and acquisition times

A nuclear physician scored each scan on the image quality using a 7-point scale (1 = very poor image quality, 7 = exceptional image quality) with the assumption of equal distances between each point. The score was based on the ability to distinguish lesions from the background. Lesions were determined based on concordance of findings with CT images and gained experience. To assess the different dosages, image quality scores were compared between the two dosage groups. To determine the optimal acquisition time and duration, the image quality score and lesion detection of patients who presented with less than 10 suspicious lesions were evaluated within each dosage group. This restriction was included to avoid disproportional impact of patients with multiple lesions on the overall result. The percentage agreement was calculated using the images at T60, 3 min/bp as the reference. Finally, the relative contrast noise ratio (CNR) was determined for all suspicious lesions observed within the 2 MBq/kg group. The relative CNR is equal to the ratio of the relative contrast and relative noise, which are calculated using Eq. 1 and 2, respectively. Relative CNR values of T60, 3 min/bp images will be compared to relative CNR values of T60, 1.5 min/bp and T180, 3 min/bp images to evaluate the effect on lesion visualization of acquisition duration and time, respectively.

$$ \mathrm{relative}\ \mathrm{contrast}=\frac{{\mathrm{SUV}}_{\mathrm{peak}\ L}-{\mathrm{SUV}}_{\mathrm{mean}\ B}\ }{\mathrm{SUV}\mathrm{peak}\ L} $$

Equation 1: Calculation of relative contrast with SUV = standardized uptake value, L = suspicious lesion and B = 10 mm bulb shell around the suspicious lesion

$$ \mathrm{relative}\ \mathrm{noise}=\frac{{\mathrm{standard}\ \mathrm{deviation}}_H}{{\mathrm{mean}}_H} $$

Equation 2: Calculation of relative noise with H = spherical region (*r* = 2 cm) in proximity of the suspicious lesion

### Evaluation of co-administration of furosemide

Because ^18^F-PSMA-11 shows a high urinary clearance (29.0 ± 5.9% 300 min p.i.) [4], a diuretic (furosemide) was added to the study protocol to reduce interference of the ureters on the PET image. To assess the effect of furosemide on the image quality, 22 additional patients were recruited. The PET images were compared to the initial 22 patients who underwent the same scan protocol with administration of furosemide. The interference of the radioactivity uptake in the bladder and the ureters on the interpretation of the scan was evaluated on a 7-point scale (1 = not disturbing, 7 = very disturbing) with the assumption of equal distances between each point.

### Interrater reliability

To evaluate the variability in the interpretation of detected suspicious lesions on the PET scans, two independent observers who were blind for dosage group but with access to the medical history assessed each PET/CT scan. Both observers had at least 4 years clinical experience as nuclear physicians and had evaluated ^18^F-PSMA-11 scans in the previously conducted Phase 1 trial. No previous study-related training in PSMA image readings was given and there was no study-related communication between the two observers during the data handling. The interrater reliability was evaluated based on images of the preferred scan time and duration. Each nuclear physician appointed the patients to one of following disease status: no tumour, local disease, locoregional disease, oligo- (0–3 suspicious lesions) or polymetastatic (≥ 4 suspicious lesions) disease. Observed suspicious lesions were described and subdivided into the following locations: prostatic, lymphatic, bone or visceral metastasis. When patients presented with more than 10 suspicious lesions, no description of the lesions was performed and these results were therefore not included in the analysis. The interrater reliability was based on concordance of disease status, the total number of suspicious lesions per location and agreement on each suspicious lesion separately. To determine if a learning curve is applicable to the reliable interpretation of images, all analyses were performed on the predetermined optimal scan protocol for both the initial and the later conducted extended Phase 2 study.

### Statistical analysis

All statistical analyses were performed using R software [7]. For comparison of ordinal data between the two groups (comparison of dosage groups, evaluation of the effect of furosemide), the Mann-Whitney *U* test was performed. For the detection of a difference between > 2 paired groups (comparison of four timeframes: T60, 1.5 and 3 min/bp, and T180, 1.5 and 3 min/bp), a pairwise Wilcoxon signed-rank test corrected for multiple testing (Bonferroni-Holm correction [8]) was performed. However, the difference in image quality was only considered clinically relevant when the difference in median value was more than 1 point. *κ* statistics and 95% confidence intervals were calculated using the ‘psych’ package [9] to evaluate the interrater reliability. The *κ* values were interpreted according to the categories presented in Table 2 [10]. The significance level for all tests was set on *α* = 0.05.

**Table 2:** Determination of interpretation categories of kappa values, adapted from Landis and Koch [10]

*κ*	Level of agreement
< 0	Poor
0.00–0.20	Slight
0.21–0.40	Fair
0.41–0.60	Moderate
0.61–0.80	Substantial
0.81–1	Almost perfect

## Results

### Administration of ^18^F-PSMA-11

Synthesis of ^18^F-PSMA-11 was performed as described by Kersemans et al. [3]. The radiochemical purity was determined using high-performance liquid chromatography and thin-layer chromatography and exceeded 98% and 96%, respectively. Of the initial 44 patients included, 41 patients completed the study protocol of which 21 and 20 subjects were part of the 2 and 4 MBq/kg group, respectively. Of the 22 patients recruited in the additional Phase 2, 21 patients completed the study protocol. Patients of the 2 MBq/kg and 4 MBq/kg group were administered 167 ± 29 MBq ^18^F-PSMA-11 (4.2 ± 2.4 μg PSMA-11) and 313 ± 56 MBq ^18^F-PSMA-11 (8.3 ± 4.1 μg PSMA-11), respectively. Patients of the additional Phase 2 study were administered 161 ± 23 MBq ^18^F-PSMA-11 (7.3 ± 3.8 μg PSMA-11). Except for one patient who suffered from an allergic cutaneous reaction at the palms (grade 2 CTCAE; likely related to the CT contrast Visipaque®), none of the patients reported any subjective side effects. Creatinine level changes showed a wide variability with an average change in serum creatinine levels of 1.67% ± 10.67%.

### Optimisation of scan protocol

**Fig. 2:**
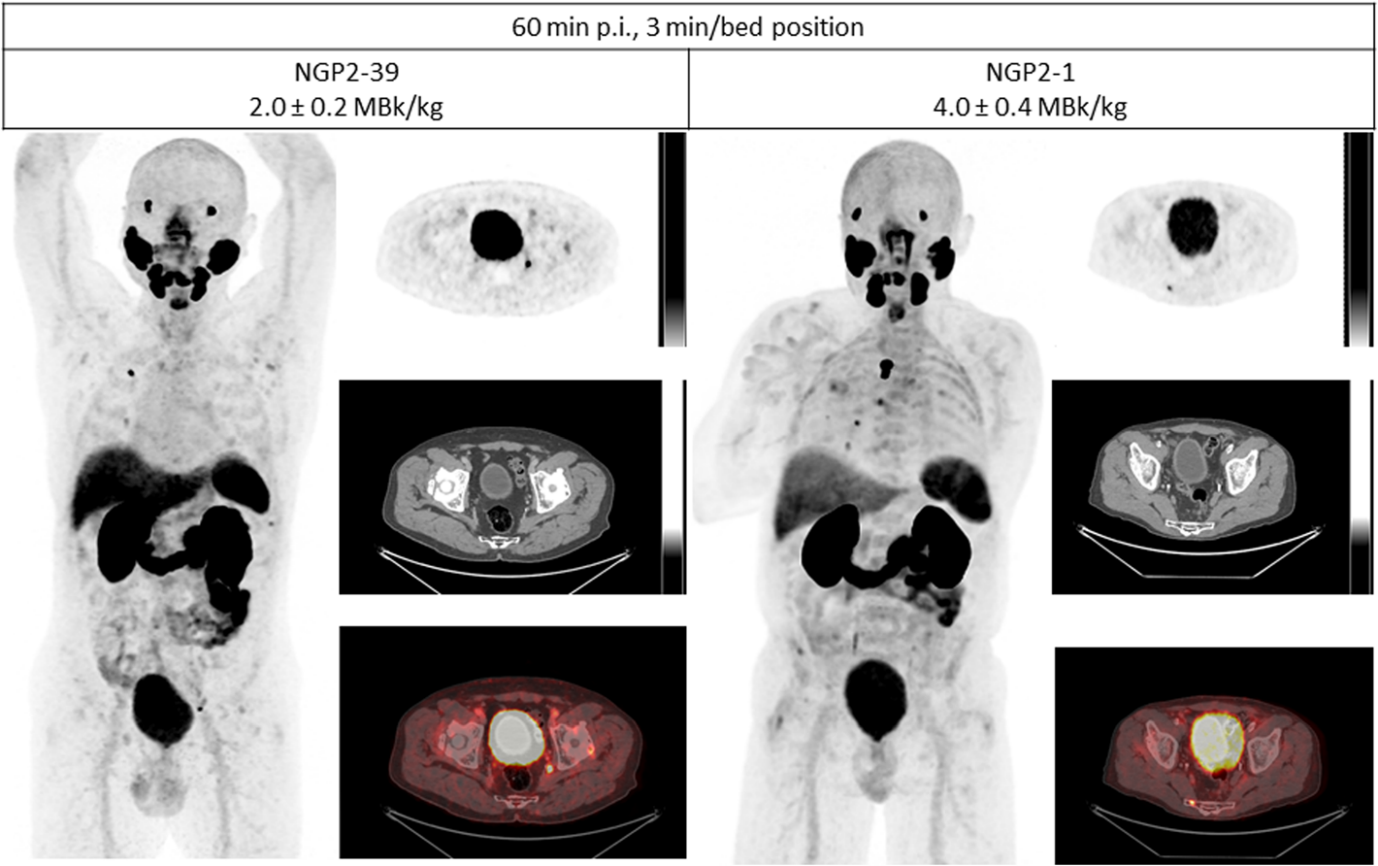
Comparison of images at T60, 3 min/bed position of two patients NGP2-39 and NGP2-1 who received 2 and 4 MBq/kg, respectively. NGP2-*x =* patient ID

The median and interquartile range (IQR) of the image quality scores are presented in Table 3 with *p* values evaluating the difference between the dosage groups for each time frame. The median scores of the 4 MBq/kg were 1 point higher (T60, 1.5 and 3 min/bp and T180, 1.5 min/bp) or equal (T180, 3 min/bp) to the 2 MBq/kg group (Fig. 2). All *p* values were below the significance level of 0.05. However, the difference in median scores between the dosage groups did not exceed the clinically relevant difference of > 1 point. A detailed overview of the results can be found in the Additional file 1.

**Table 3:** Median, interquartile range (IQR) and *p* values of the image quality scores of the 2 and 4 MBq/kg groups for sets of reconstructed images (60 and 180 min p.i., 1.5 and 3 min scan time per bed position)

	2 MBq/kg	4 MBq/kg	*p* value
	Median (IQR)	Median (IQR)	
T60, 1.5 min/bp	4 (0)	5 (1)	0.001132*
T60, 3 min/bp	5 (1)	6 (1)	0.007741*
T180, 1.5 min/bp	1 (1)	2 (0.5)	0.0007*
T180, 3 min/bp	3 (1)	3 (0.25)	0.001987*

*p values < 0.05 were considered statistically significant

*bp* bed position

**Fig. 3:**
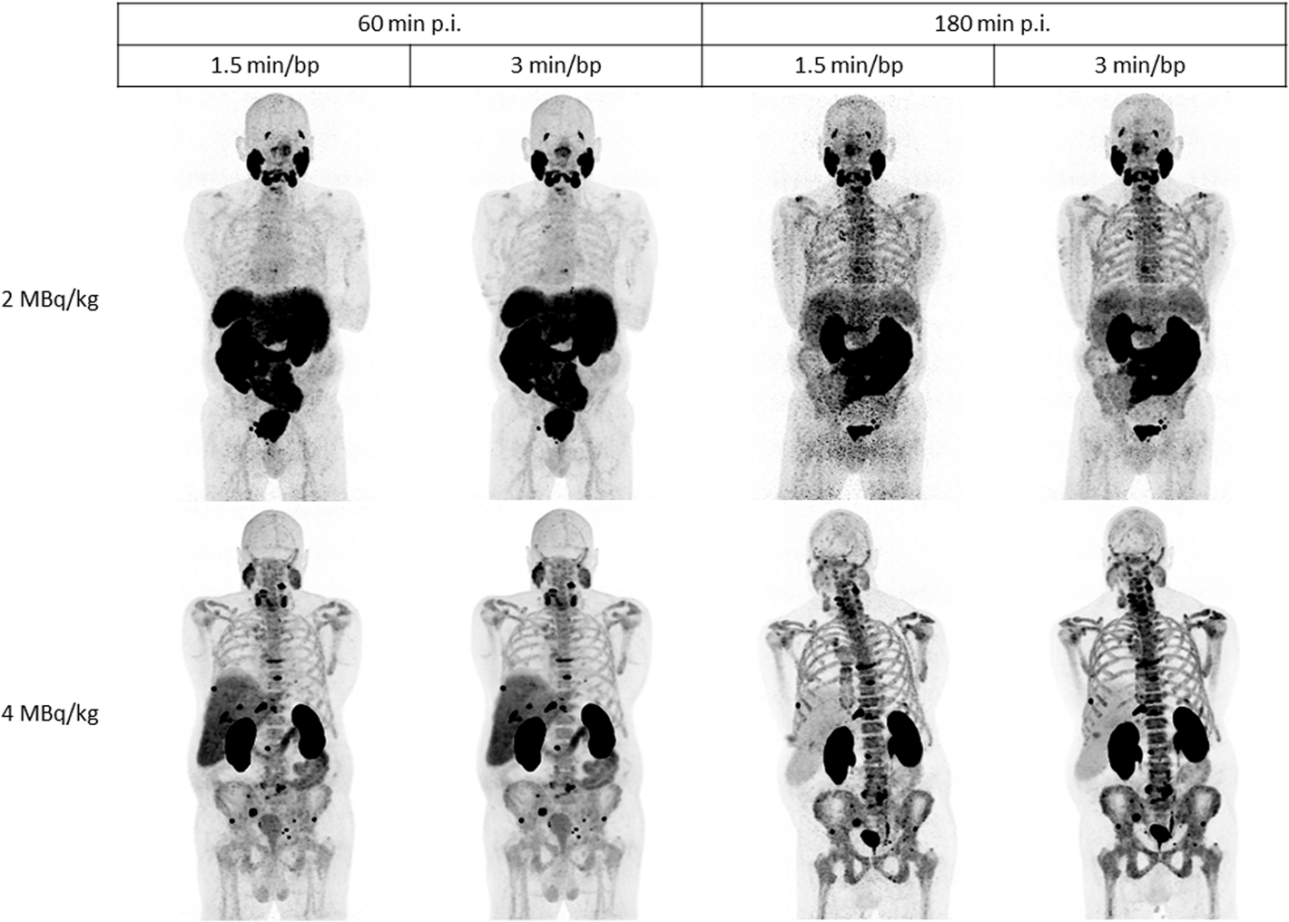
MIP images of patients NGP2-6 (2 MBq/kg, top) and NGP2-4 (4 MBq/kg, bottom). NGP2-*x* = patient ID

**Fig. 4:**
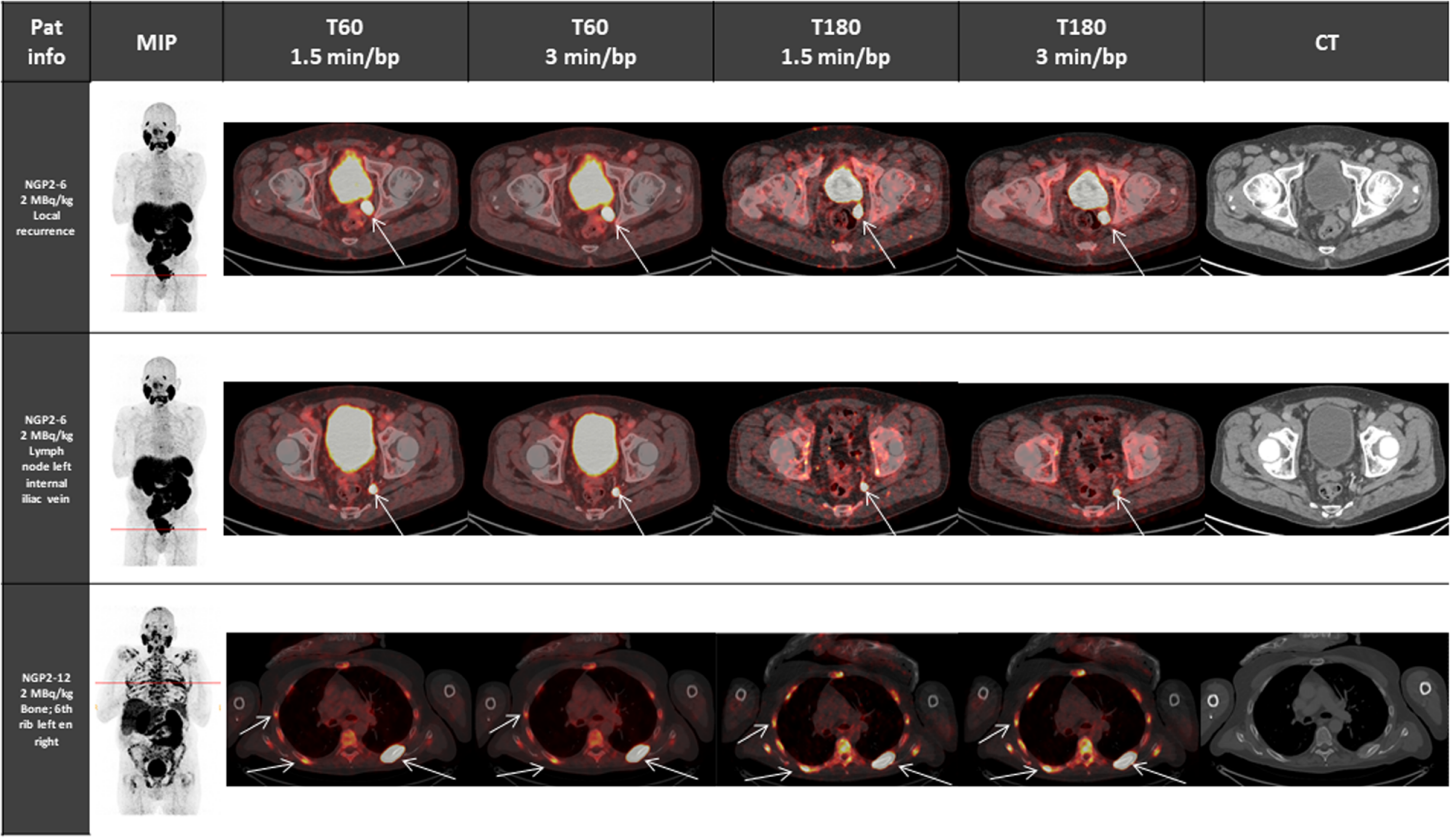
Overview of patient images who were administered 2 MBq/kg who presented with common lesion locations: local recurrence (first row), lymph node metastases (middle row) and bone metastases (last row), each presented as a MIP image (at T60, 3 min/bp) followed by axial images (axis line displayed on MIP image) of all reconstructed time frames (T60, 1.5 and 3 min/bp and T180, 1.5 and 3 min/bp), and the high dose CT image

**Fig. 5:**
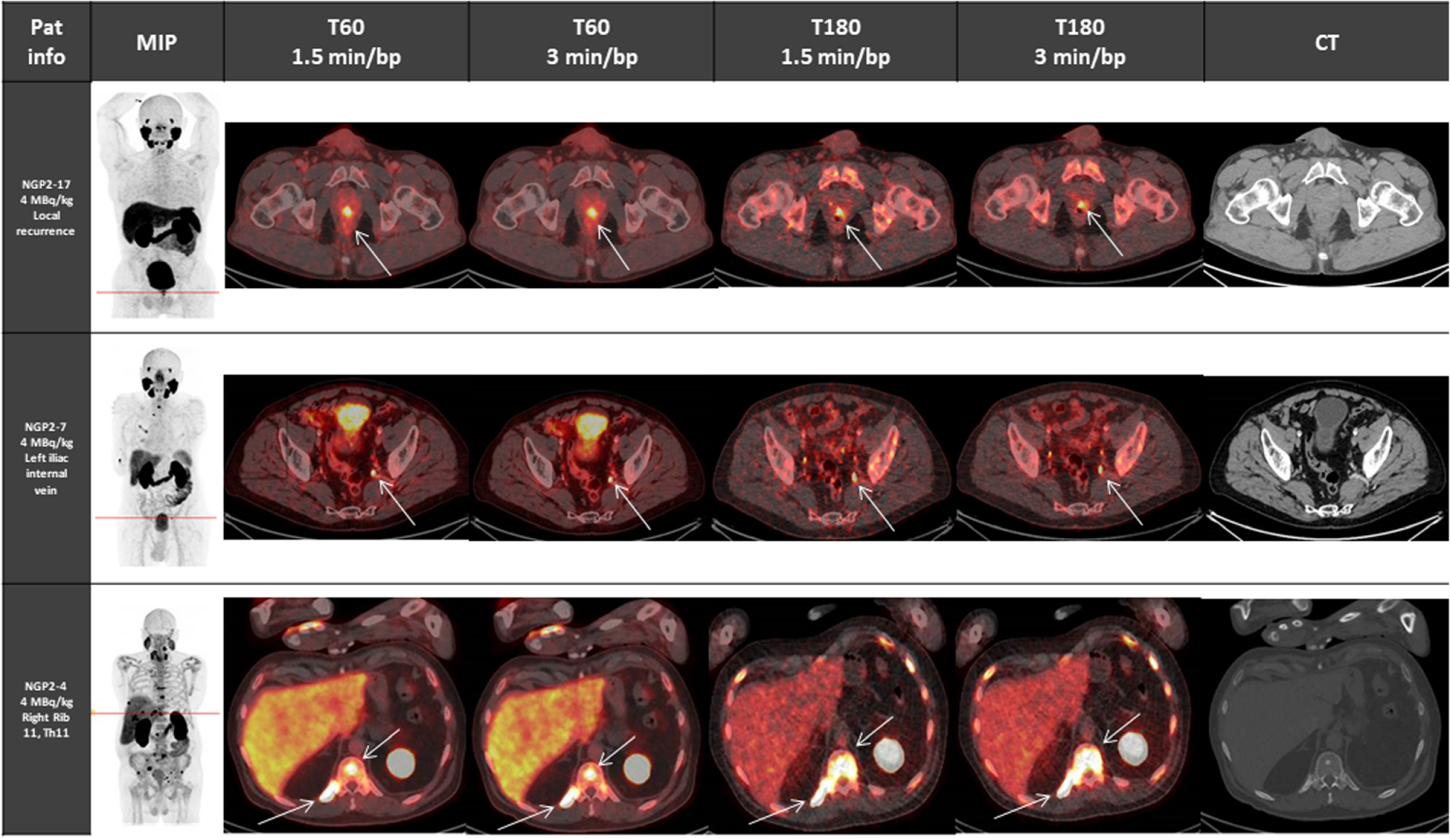
Overview of patient images who were administered 4 MBq/kg who presented with common lesion locations: local recurrence (first row), lymph node metastases (middle row) and bone metastases (last row), each presented as a MIP image (at T60, 3 min/bp) followed by axial images (axis line displayed on MIP image) of all reconstructed time frames (T60, 1.5 and 3 min/bp and T180, 1.5 and 3 min/bp), and the high dose CT image

For the evaluation of the optimal scan time (T60 vs T180) and scan duration (1.5 versus 3 min/bp), all sets of reconstructed images were compared to one another within each dosage group. In general, most images at T180 showed high background noise and were difficult to read (Fig. 3). The images at T60, 3 min/bp showed the highest median image quality score in both dosage groups (5 and 6 for 2 and 5 MBq/kg, respectively). Table 4 shows that the pairwise comparison of the image quality scores between all sets of reconstructed images was significantly different from each other for both dosage groups (*p* values < 0.05). This suggested that imaging 60 min p.i. for 3 min per bed position provides the best image quality. Pairwise comparison of suspicious lesions between all sets of reconstructed images only showed a significant difference in the 2 MBq/kg group between T180, 1.5 min/bp images and all other scan times (Table 5). The percentage agreement between suspicious lesions detected on the reference scan T60, 3 min/bp for 2 and 4 MBq/kg and comparison scans were 90.5% and 96.9% for the T60, 1.5 min/bp scan, 42.9% and 61.5% for the T180, 1.5 min/bp scan and 71.4% and 60% for the T180, 3 min/bp scan, respectively. The relative CNR of corresponding suspicious lesions of 2 MBq/kg images on T60 between 1.5 min/bp and 3 min/bp increased 16.4% (95% CI, 11.3–21.5). When comparing corresponding suspicious lesions on T60, 3 min/bp and T180, 3 min/bp images, the relative CNR decreased over time by 31.0% (95% CI, 20.8–41.3). A comparative overview of representative lesions between dosage groups and reconstructed time frames is given in Figs. 4 and 5.

**Table 4:** *p* values of the pairwise comparison of the image quality scores within the 2 MBq/kg group and the 4 MBq/kg group for all sets of reconstructed images. *p* values < 0.05 were considered statistically significant

	T60, 1.5 min/bp	T60, 3 min/bp	T180, 1.5 min/bp
Image quality score 2 MBq/kg			
T60, 3 min/bp	0.00038	–	0.00027
T180, 1.5 min/bp	0.00024	0.00027	–
T180, 3 min/bp	0.00074	0.00038	0.00047
Image quality score 4 MBq/kg			
T60, 3 min/bp	0.00011	–	0.00026
T180, 1.5 min/bp	0.00026	0.00026	–
T180, 3 min/bp	0.00026	0.00025	0.00021

*bp* = bed position

**Table 5:** *p* values of the pairwise comparison of the lesion detectability within the 2 MBq/kg group and the 4 MBq/kg group for all sets of reconstructed images. *p* values < 0.05 were considered statistically significant

	T60, 1.5 min/bp	T60, 3 min/bp	T180, 1.5 min/bp
Detection of suspicious lesions 2 MBq/kg			
T60, 3 min/bp	1	–	0.0063
T180, 1.5 min/bp	0.0063	0.0063	–
T180, 3 min/bp	1	1	0.0063
Detection of suspicious lesions 4 MBq/kg			
T60, 3 min/bp	1.00	–	0.17
T180, 1.5 min/bp	0.17	0.17	–
T180, 3 min/bp	0.21	0.21	1.00

*bp* = bed position

### Evaluation of co-administration of furosemide

The median score of the interference on the bladder and ureters was 2 (IQR = 0) for the furosemide group and 3 (IQR = 2) for the group without administration of furosemide (*p* value of 0.01106 (< 0.05)). This suggests slightly less interference when furosemide was administered. Although statistically significant, the median difference of 1 is lower than the predetermined clinically relevant difference of more than 1 point.

### Evaluation of interrater reliability

**Fig. 6:**
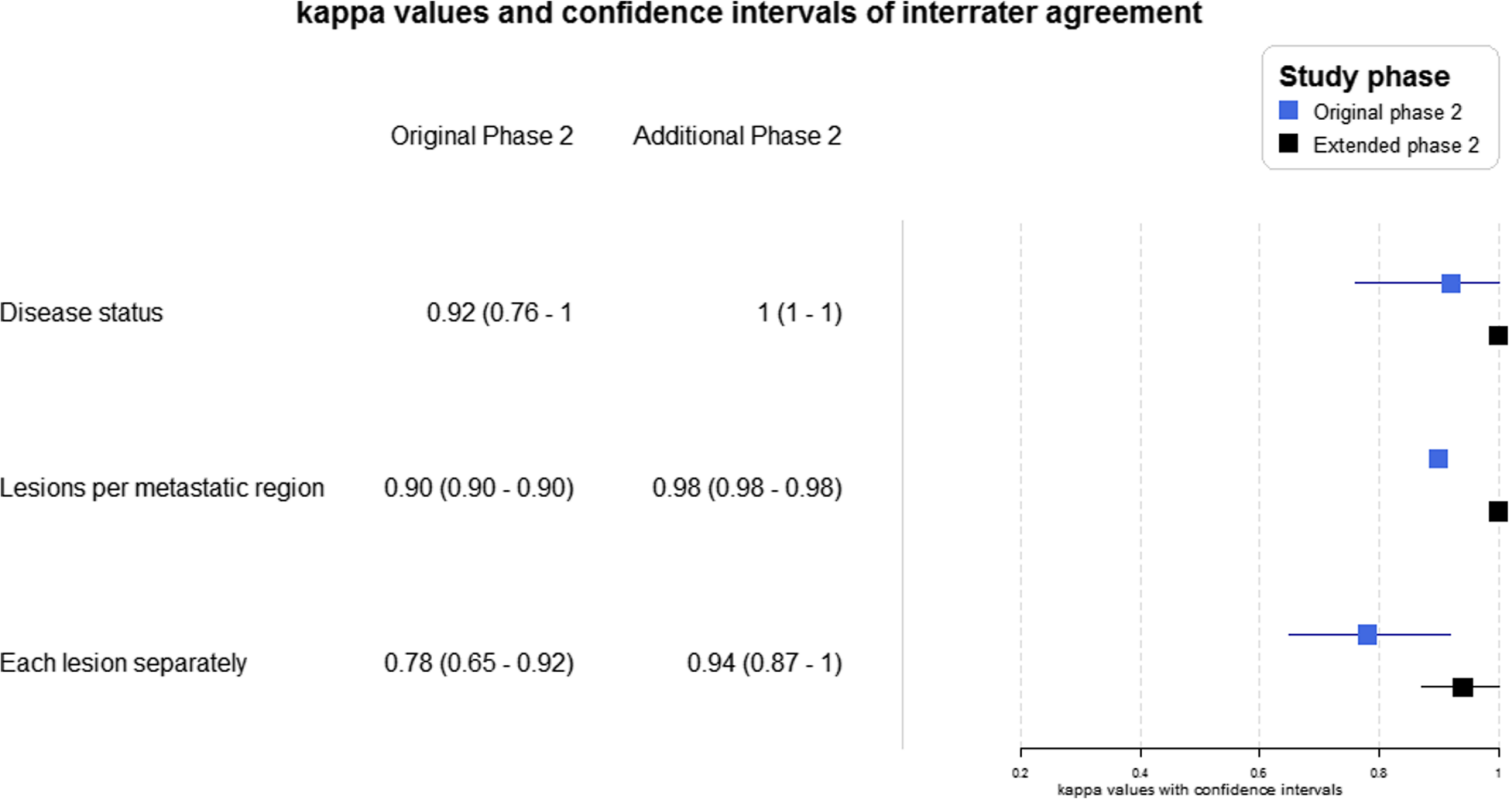
Comparison of interrater reliability kappa statistics between the original Phase 2 study and the additional Phase 2 study

All analysis was performed on the T60, 3 min/bp scans of the 2 MBq/kg group. When considering the agreement in disease status (no tumour, local, locoregional, oligo- or polymetastatic), the *κ* value of 0.92 (95% CI, 0.76–1) suggests an almost perfect interrater agreement. For the ability to detect suspicious lesions reliably in the most common metastatic regions (prostate, lymph nodes, bone and viscera), an overall *κ* value of 0.90 (95% CI, 0.90–0.90) is found. The comparison of each lesion separately gives a *κ* value of 0.78 (95% CI, 0.65–0.92) which indicates a substantial agreement. To evaluate if the interrater reliability increases after gaining experience, the same analyses were performed for the patients of the extended Phase 2. The *κ* statistics for the agreement in disease status, metastatic regions and lesions separately were 1 (95% CI, 1–1), 0.98 (95% CI, 0.98–0.98) and 0.94 (95% CI, 0.87–1). The comparison of the results is presented in a forest plot (Fig. 6).

## Discussion

An increased interest in PSMA-based radiotracers has led to multiple clinical trials on PSMA PET imaging for prostate cancer [11–13]. To achieve an accurate and reliable image interpretation, a standardized scan protocol is necessary for implementation in clinical practice [14]. This study determines the optimal scan protocol for PET imaging of ^18^F-PSMA-11 by considering the effect of activity dosage, acquisition time, scan duration and furosemide co-administration on image quality and lesion detection.

Various dosage schemes have been proposed for PSMA radiotracers. For ^68^Ga-PSMA-11, the joint guidelines of EANM and SNMMI recommend a dosage of 2.0 ± 0.2 MBq/kg [14], while for ^18^F-PSMA-1007, dosages up to 4 MBq/kg are applied [15]. In this study, two dosage groups (2 and 4 MBq/kg body weight) were compared. The administered activity is the primary factor that determines the radiation exposure and should therefore be kept as low as reasonably achievable (ALARA principle) while minimizing the loss of diagnostic information [16–18]. The higher dosage of 4 MBq/kg and associated increased radiation exposure will therefore only be tolerated when the difference in median score between both dosage groups is more than 1 point, as smaller differences will not be considered clinically relevant. Although the median difference between the two dosage groups was statistically significant, the > 1 point difference criterion was not achieved to accept the 4 MBq/kg body weight as dosage. Therefore, 2.0 ± 0.2 MBq/kg body weight is recommended for achieving images of sufficient quality while limiting the radiation exposure. This is in agreement with the joint EANM and SNMMI guidelines for PET/CT imaging with ^68^Ga-PSMA [14]. For dosage optimization, inter-subject comparison was not possible as this would imply administration of both doses to each patient, which would unnecessarily increase patient radiation exposure. A compromise for this limitation is the comparison of the lesion detection of patients injected with 4 MBq/kg between the short (1.5 min/bp) and long (3 min/bp) scan time per bed position. One can argue that assuming linearity of the PET camera, imaging at half the acquisition time can simulate the administration of half the dosage. As seen in Table 5, the *p* value of the comparison between T60, 1.5 and 3 min and T180, 1.5 and 3 min is both times equal to 1, which confirms the recommendation of a dosage of 2 MBq/kg ^18^F-PSMA-11.

Many studies have compared early and delayed scanning; however, opposite results were obtained. Schmuck et al. and Afshar-Oromieh et al. both found a higher tumour-to-background ratio for some lesions on delayed ^68^Ga-PSMA-11 imaging but did not agree on the change in overall detection rate [19, 20]. Rahbar et al. found no additional benefit in delayed ^68^Ga-PSMA-11 acquisition [21], while Derlin et al. showed an improved image quality for ^68^Ga-PSMA-I&T on delayed imaging but only when combined with delayed furosemide administration [22]. This shows that the acquisition time can be an important scan parameter but no consensus has yet been reached on this topic. As the longer half-life of ^18^F makes delayed imaging more accessible, it was deemed appropriate to evaluate imaging 1 h and 3 h after ^18^F-PSMA-11 administration. Images at T180 were more difficult to read and there appears to be increased uptake in the bone. However, a decrease in CNR was observed over time. This suggests that either the uptake in surrounding bone tissue increases and/or lesion uptake decreases. Additional analysis of the Phase 1 study [4] showed that the activity in bone versus total body increased with 10.86% from 90 to 300 min p.i. This increase is probably partially caused by free 18F. However, 90 min p.i., less than 10% of the injected activity is present in the blood of which only 22.2 ± 1.5% is free ^18^F-fluoride. This means that the amount of ^18^F-fluoride in absolute quantity is low. Another contributing factor could be slow tracer clearance from bone tissue, but this should be further investigated. This observation corresponds to a study by Afshar-Oromieh et al. who evaluated additional late scanning with ^68^Ga-PSMA-11 [20]. Images at T60, 3 min/bp were given the highest median image quality scores and were therefore chosen as reference for following analyses. For the number of detected suspicious lesions, only T180, 1.5 min/bp was significantly inferior. Since only 42 lesions were detected in 11 patients, the number of lesions might be too small to detect a statistical difference between the other scan images. A high percentage agreement was only found between images at T60 (90.5%) while fewer lesions were detected on images at T180. Although the total number of detected suspicious lesions on T180, 3 min/bp were initially similar to the reference scan (32 vs 34 lesions respectively), retrospective analysis of the detected suspicious lesions showed that the suspicion of certain bone lesions was due to low image quality or aspecific uptake because of degenerate bone disease, which reduced the total number from 32 to 28. Finally, the relative CNR of suspicious lesions increased for longer acquisition times while decreasing when scanning occurred at a later time point. Taking into account all the abovementioned results, imaging 60 min p.i. with a scan duration of 3 min per bed position was recommended, which is in accordance to the joint EANM and SNMMI guidelines on imaging with ^68^Ga-PSMA [14]. However, despite the lower image quality, the similar lesion detection rate of T60, 3 min/bp and T60, 1.5 min/bp would make the latter a possible acquisition scheme when shorter acquisition times are required. These results confirm similar previous findings by Goethals et al. [23] and Hausmann et al. [24]. No evidence was found to recommend delayed imaging with ^18^F-PSMA-11 in our study. No dynamic whole-body PET scans were performed for several reasons. Firstly, the previously conducted Phase 1 study [4] showed no increased uptake in major organs (except the kidneys) or possible lesions between 50 and 90 min p.i. Also, a broad set of inclusion criteria was applied to obtain a diverse cohort of patients in order to evaluate the radiotracer for different disease stages and metastases locations. Therefore, the condition of many elderly patients did not allow for intensive dynamic imaging protocols. Furthermore, the administration of a diuretic prevented prolonged scan duration. Therefore, the study protocol was limited to two acquisition times based on previously reported results [4] and literature reporting on scanning 60 min p.i. and increased lesion detectability 180 min p.i [14, 25, 26]. Dynamic imaging will be further investigated in ongoing animal experiments.

Furosemide co-administration reduced slightly the radiotracer interference on the ureters. However, the clinically relevant median difference of > 1 point was not achieved. This means that a good image quality can be obtained without the need for a diuretic which can cause additional discomfort. Nonetheless, the addition of furosemide can be beneficial for the detection of lymph nodes in the prostate region or in proximity of the ureters [22]. In this study protocol, a rather high dose of furosemide (40 mg) was administered 30 min before the start of the first PET/CT. It may be more appropriate to administer a lower furosemide dose simultaneously with the radiotracer administration for the comfort of the patient, which is also recommended in the guidelines of EANM and SNMMI for administration of ^68^Ga-PSMA [14].

PSMA expression is not specific to the prostate only; physiological background uptake is seen in the kidneys, bladder, intestines, and salivary glands as well as in pathological conditions such as Paget’s bone disease and neovasculature of several solid tumours such as high-grade sarcomas [27–31]. Moreover, some prostatic malignant lesions exhibit minimal PSMA expression and can be difficult to detect on PET [32]. Therefore, the interrater reliability can be a useful parameter to assess the clarity of ^18^F-PSMA-11 image interpretation and the potential need for gaining experience in differentiating prostate cancer lesions from benign focal uptake spots. The interrater reliability was assessed twice. Initially, the grade of agreement was determined in the original Phase 2 study within the 2 MBq/kg group on T60, 3 min/bp images. To see if a learning curve is applicable on the interpretation of ^18^F-PSMA-11 images, the analysis was repeated on images of the additional Phase 2 study which were carried out after all images from the initially included patients were evaluated. It was considered valuable to evaluate the ability to assign the correct disease status (local, locoregional, oligo- and polymetastatic disease) to each patient as this plays an important role in determining the appropriate treatment. The *κ* statistic of 0.92 (95% CI, 0.76–1) suggested an almost perfect interrater agreement in disease status and even increased to 1 (95% CI, 1–1) for the additional Phase 2. This would make ^18^F-PSMA-11 an important tool for knowing the disease status of the patient, which could influence the treatment management plan. The forest plot in Fig. 6 clearly shows improvement in each category where *κ* statistics were determined. This was in concordance with the interobserver agreement study by Fendler et al. [27] who also found that observers with less than 30 previous PSMA image readings only showed moderate interrater reliability contrary to more experienced observers who achieved substantial to almost perfect agreement. Retrospectively, all observed lesions from the original Phase 2 were discussed with both observers. Of the seven bone lesions on which the observers did not agree, two bone lesions were determined suspicious and subjected to follow-up while the other five bone lesions would not be made suspicious again after the gained experience from the trial.

This study was accompanied by some limitations. Firstly, determining the optimal dosage was solely based on a subjective scoring system. Furthermore, the PET characteristics also play a role in scan parameters. This was not accounted for as all scans were performed on one PET system (GE Healthcare). Finally, further optimization of image reconstruction parameters such as number of subsets, number of iterations and post-smoothing kernel should be investigated.

## Conclusion

The best image quality was obtained by administration of 4.0 ± 0.4 MBq/kg ^18^F-PSMA-11. However, preference will be given to a dosage of 2.0 ± 0.2 MBq/kg due to the small difference in absolute score (max 1 point) and the ALARA principle. PET acquisition should start 1 h post injection with 3 min per bed position acquisition time. Depending on the medical history of the patient and the location of metastatic lesions, the co-administration of a diuretic can be useful. The increase of the *κ* value from 0.78 to 0.94 suggests that the interpretation of ^18^F-PSMA-11 is more reliable after 40+ PSMA scan readings.

## Supplementary information

**Additional file 1.** Dataset representing detailed results of the Phase 2 clinical trial study on ^18^F-PSMA-11.

## Data Availability

The datasets generated during the current study are available from the corresponding author on reasonable request.

## Abbreviations

^18^F-PSMA-11: 18-fluorine prostate-specific membrane antigen 11
ALARA: As low as reasonably achievable
bp: Bed position
CI: Confidence interval
CNR: Contrast noise ratio
IQR: Interquartile range
p.i.: Post injection
PSMA: Prostate-specific membrane antigen
T180: 180 min post injection of the radiotracer
T60: 60 min post injection of the radiotracer
